# A comparative study in learning curves of laparoscopic lateral suspension vs. laparoscopic sacrocolpopexy: preliminary results

**DOI:** 10.3389/fsurg.2023.1274178

**Published:** 2023-12-06

**Authors:** Ewelina Malanowska-Jarema, Yana Osnytska, Andrzej Starczewski, Matteo Balzarro, Emanuele Rubilotta

**Affiliations:** ^1^ Department of Gynecology, Endocrinology and Gynecologic Oncology, Pomeranian Medical University, Szczecin, Poland; ^2^ Department of Urology, Azienda Ospedaliera Universitaria Integrata Verona, Verona, Italy

**Keywords:** prolapse, laparoscopic surgery, sacropexy, learning curve, lateral suspension

## Abstract

**Background:**

Determination of the learning curve of new techniques is essential to improve safety and efficiency. Limited information is available regarding learning curves of different techniques in laparoscopic pelvic floor surgery.

**Objective:**

The aim of this study was to compare the learning curve of two operative techniques, laparoscopic lateral suspension (LLS) and laparoscopic sacrocolpopexy (LSC).

**Material and methods:**

We conducted a prospective study to assess the learning curve of LLS and LSC by implementing a structured urogynecologic surgical training program with the use of pelvic trainers for our urogynecology fellow. The fellow was an experienced urogynecologic surgeon, but was laparoscopic suturing and dissection naive at the beginning of the study. She was required to assist in 20 laparoscopic urogynecologic surgeries and undertake laparoscopic suturing and knot tying training with mesh positioning on a laparoscopic trainer for 4 h/week during the trial period. After the completion of this structured training program, the fellow performed LLS and LSC under the supervision of an experienced subspecialist as the primary surgeon. Linear regression analysis was used to compare the data of LLS and LSC learning curves. Subjective pre- and post-operative evaluation of pelvic organ prolapse (POP) and pelvic floor disorders was undertaken preoperatively and 12 months postoperatively using the PFDI-20-Quality of Life validated questionnaire. Follow-up was scheduled 12 months after the surgery and performed by a skilled urogynecologist. Objective cure was defined as Pelvic Organ Prolapse-Qualification (POP-Q) stage <II in any compartment.

**Results:**

The mean operative times of laparoscopic sacrocolpopexy and lateral suspension were 168.26 and 160.33 min, respectively. According to linear regression analysis after 43 procedures, the learning curve for laparoscopic lateral suspension was shorter than for laparoscopic sacrocolpopexy (OPTime 134.69 min). In both groups, there was a significant reduction in bothersome POP symptoms (*p* ≤ 0.005). Bladder injuries in two cases and lumbar pain in one case were recorded during the study. Overall objective success at 12 months was 90.7% for LSC and 89.1% for LLS.

**Conclusion:**

Laparoscopic lateral suspension could be an alternative to laparoscopic sacrocolpopexy in the treatment of POP with its good objective and subjective outcomes. Lateral suspension has a shorter learning curve, and it is technically less demanding than LSC. Procedure-dedicated training can accelerate the move from a *novice* to a master laparoscopic surgeon.

## Introduction

Pelvic floor disorders are common, but still an underestimated problem, with detrimental effects on the quality of women's life (1). The treatment for pelvic organ prolapse (POP) should be based on the individual patient's health condition, but above all, they should depend on the given symptoms and the presence of anatomical type of pelvic floor dysfunction (2, 3). Although the majority of POP cases do not require surgical correction, the lifetime risk of undergoing surgery for genital prolapse is 12.6% (4). The frequency of performing procedures increases with age.

Surgical treatment offers a wide range of techniques including laparoscopic, abdominal, and vaginal approach in the treatment of apical defect (5). However, minimally invasive surgery provides many advantages for the patient including reduced intraoperative bleeding, shorter hospitalization, less postoperative pain, and lower rates of postoperative wound infection (6).

Abdominal sacrocolpopexy, which was first described and performed via laparotomy, has been replaced laparoscopically, which showed similar anatomical outcomes but lower complication rates in many studies (7–9). Sacrocolpopexy is considered the gold standard for the repair of apical defects. However, the procedure is technically fairly demanding and requires advance skills from the surgeon (10, 11). Recently, laparoscopic lateral suspension (LLS) described by Dubuisson et al. has gained much attention (12). In the available literature, results proved the efficacy of this method in the treatment of isolated apical defect with concomitant cystocele, both in anatomical and quality of life outcomes (12, 13).

Laparoscopic suturing, anatomical abnormalities, and challenges in dissection of the promontorium may cause fear and delay in implementing such techniques in fellowship programs. However, the growing number of women with POP, who prefer laparoscopy, leads to bigger interest in surgical training programs in the field of urogynecology (14).

Learning curves are important in quality improvement initiatives as they help identify critical points in the learning process where errors and complications are more likely to occur.

This knowledge can lead to better patient outcomes and safer surgical practices (15).

The aim of the current study was to evaluate learning curve of a senior urogynecologic surgeon performing laparoscopic sacrocolpopexy (LSC) and LLS in a special dedicated training program.

## Material and methods

We conducted a prospective study to assess the learning curve of LLS and LSC by implementing a structured urogynecologic surgical training program for our urogynecology fellow.

The fellow was an experienced urogynecologic surgeon, but was laparoscopic suturing and dissection naive at the beginning of the study.

She was required to assist in 20 laparoscopic urogynecologic surgeries and undertake laparoscopic suturing with mesh placement on a laparoscopic trainer for 4 h/week during the trial period.

After completion of this structured learning program, the fellow began performing urogynecologic procedures as the primary surgeon under the supervision of a urogynecology subspecialist.

### Surgical training steps and laparoscopic trainer setting

The suturing practice was carried out on a simple LAPARO Aspire Pelvic Trainer (Trainer with the built-in HD 0° camera and LED lighting) without simulation software.

**Figure 1:**
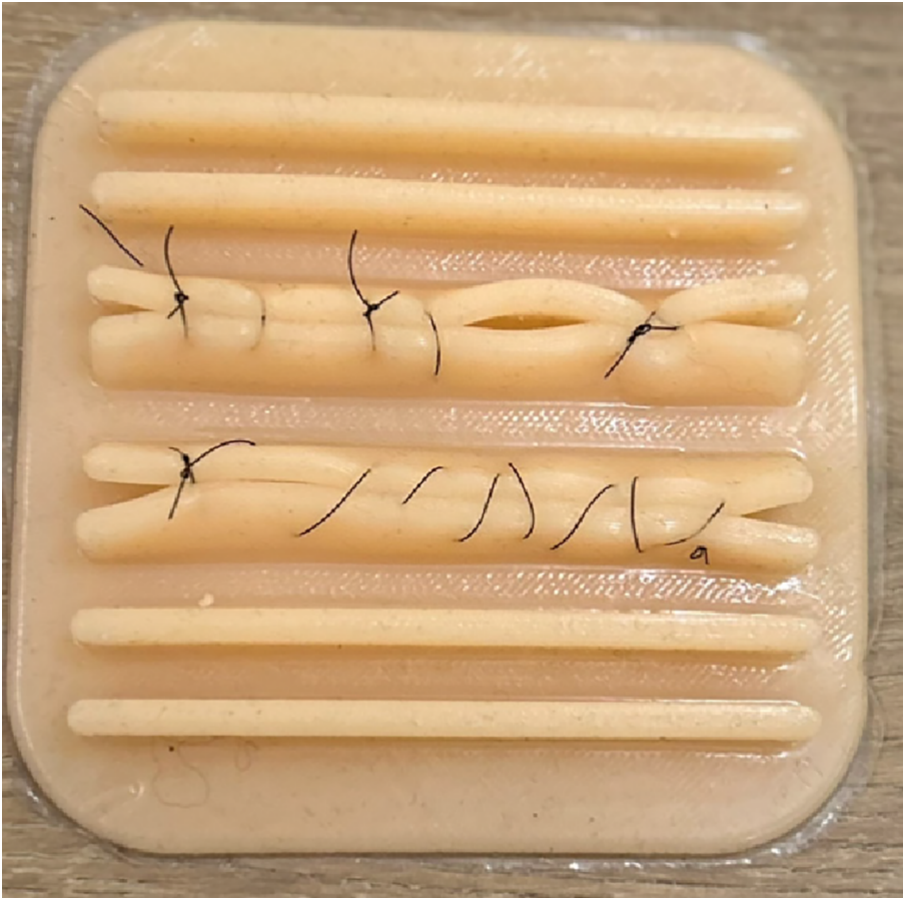
Knot tying. This task involved the tying of an intracorporeal knot and continuous suture on a silicone 3D model.

**Figure 2:**
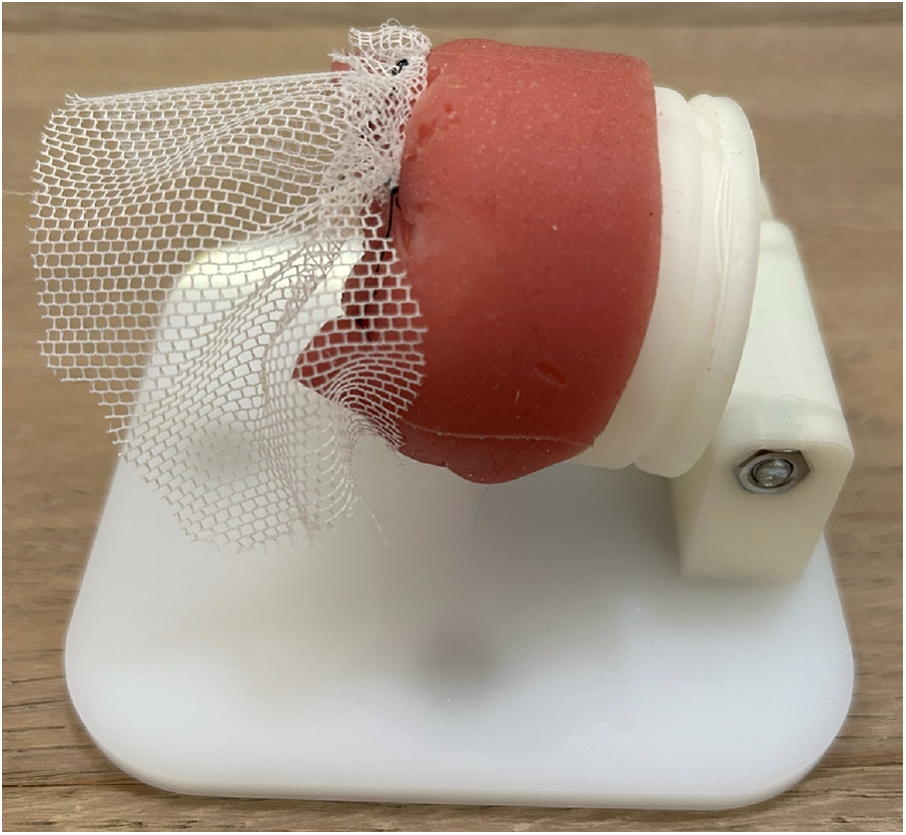
Suture placement. This task involved the mesh suture fixation on a cervix/vagina model.

**Figure 3:**
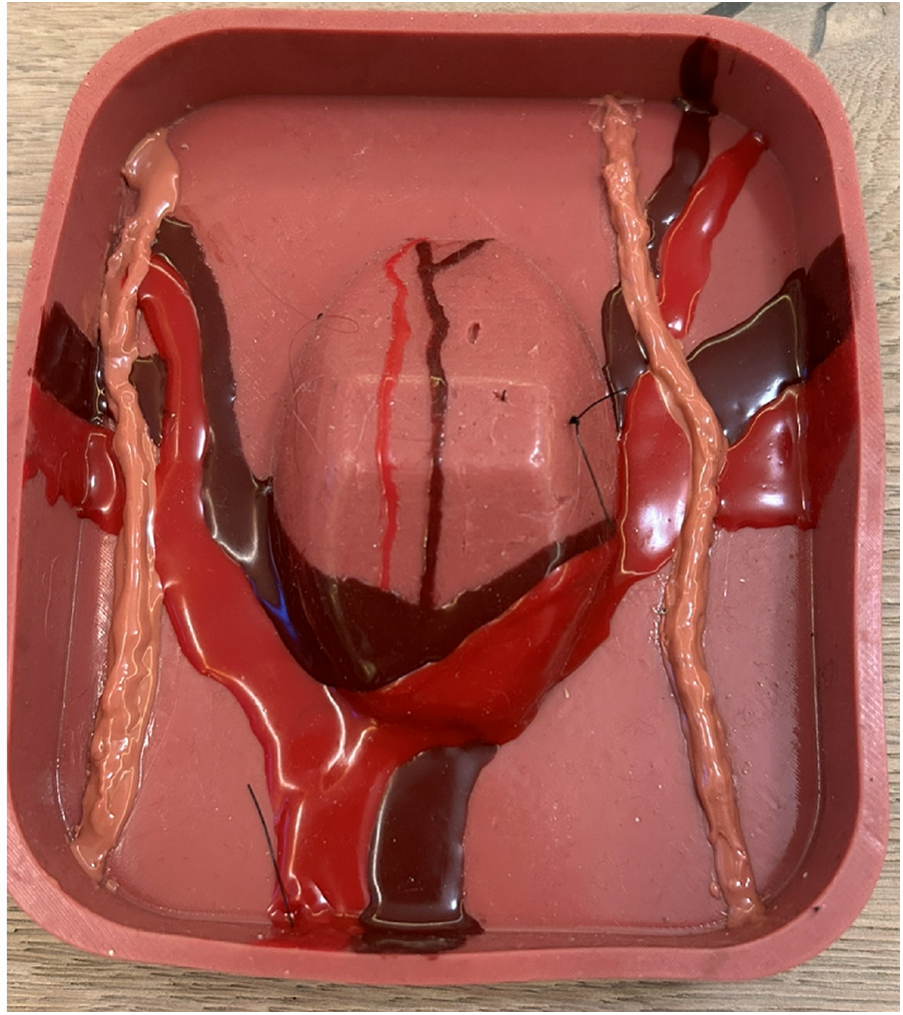
Mesh positioning on the promontory. This task required the participant to attach the mesh at the level of the promontory.

The fellow was required to use dedicated urogynecologic training models (Figures 1–3).

The training included single knot tying, continuous suturing, and mesh positioning on the promontory or cervix/vagina. The aim of the training was for the fellow to attain assured familiarity with the steps of the operation.

### Patient's criteria

This prospective study included all women consecutively referred to our department, with symptomatic stage II Pelvic Organ Prolapse-Quantification System (POP-Q) or greater apical prolapse with or without anterior compartment prolapse. A total of 22 patients had a third-degree apical prolapse and 21 had a second-degree apical prolapse before the LSC surgery. Twenty patients had a third-degree apical prolapse and 26 had a second-degree apical prolapse before LLS surgery.

Exclusion criteria were previous urogynecologic procedures, cervical pathologies, previous urogynecologic operations, neurological diseases, associated posterior vaginal wall defects, and stress urinary incontinence. Post-micturition trans-vaginal ultrasonography was performed to assess the post-void residual (PVR) of urine. All the patients underwent urodynamic testing before the surgery to detect clinical or unmasked clinical urinary incontinence by treatment of prolapse.

Subjective pre- and post-operative evaluation of pelvic organ prolapse and pelvic floor disorders was undertaken preoperatively and 12 months postoperatively using the PFDI-20-Quality of Life validated questionnaire.

Follow-up was scheduled 12 months after the surgery and performed by a skilled urogynecologist (EM). Objective cure was defined as POP-Q stage <II in any compartment.

The study was approved by the Ethics Committee on Clinical Studies of Pomeranian Medical University (KB-0012/27/17).

### Surgical technique

All women underwent laparoscopic supracervical hysterectomy with or without concomitant prophylactic salpingo-oophorectomy (women over 60 years) after reading and signing the informed consent.

Laparoscopic sacropexy was performed with one strap mesh. Peritoneal incision and dissection started at the level of promontory and was carefully extended along the rectosigmoid to the uterine cervix. The mesh was fixed with four single non-absorbable sutures to the anterior vaginal wall and cervix. The mesh was fixed to the promontory with two non-absorbable sutures (Ethibond 0). Peritoneum was then closed with a running suture (Vicryl 3-0).

A T-shaped polypropylene mesh was used for the lateral suspension. The body of the mesh was fixed to the uterine cervix and to the upper part of the anterior vaginal wall. The arms were introduced retroperitoneally toward the lateral abdominal walls, alongside round ligaments. Both arms were attached laterally to the abdominal fascia. Mesh peritonization was routinely performed.

### Statistical analysis

The data were statistically analyzed using Gretl software version 2017a. For comparison between LLS and LSC preoperatively and 12 months postoperatively, *p*-value was obtained using the *T*-test. For comparison between LLS preoperatively and 12 months postoperatively, *p*-value was obtained using the chi-square test. To compare data of LLS and LSC learning curves, linear regression analysis was used. The significance level was assumed to be *p* < 0.005.

## Results

In the first 12 months of her fellowship position, between January 2021 and January 2022, the fellow performed 89 LSCs as the primary surgeon. The consultant urogynecologist assisted more than 50% of surgeries. The mean age of the patients was 59.49 (±8.84) years, and the median body mass index was 25.98 (±3.71) kg/m^2^. Preoperatively, All women had a leading prolapse of at least POP-Q stage 2. The success rate of apical compartment prolapse was similar in both groups, and it was 90.7% for LSC and 89.1% for LLS.

Only four of the 22 patients in the LSC group with initial third grade of apical POP had symptomatic recurrence. In three patients after LLS with preoperative advanced apical POP grade III and II, we observed the recurrence or prolapse to the initial stage.

We did not find significant recurrence of posterior compartment prolapse in both groups. Only three patients, one from the LSC group and two from the LLS group had grade II of posterior compartment POP after 1 year of follow-up.

During the trial, there were bladder injuries in two cases and lumbar pain in one case after the surgery.

The conversion to laparotomy was necessary in three women: two patients who underwent sacropexy and one in the lateral suspension group. Mean blood loss was 100 ml.

Objective success at 12 months was 90.7% for LSC and 89.1% for LLS.

In both groups, there was a significant reduction in bothersome POP symptoms (*p* ≤ 0.005) with the PFDI-20-Questionnaire.

### Learning curve

**Figure 4:**
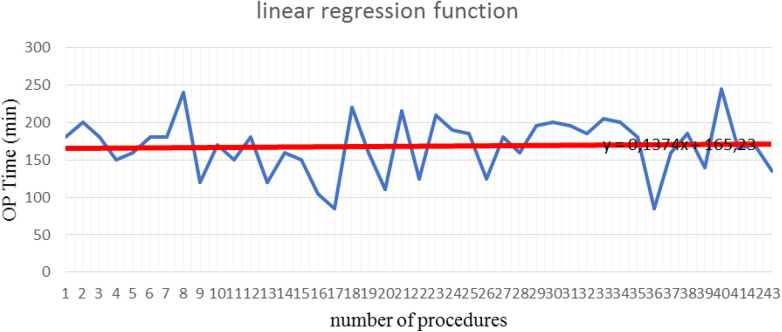
Learning curve after sacrocolpopexy (Max OPTime: 245 min, Min OPTime: 85 min).

**Figure 5:**
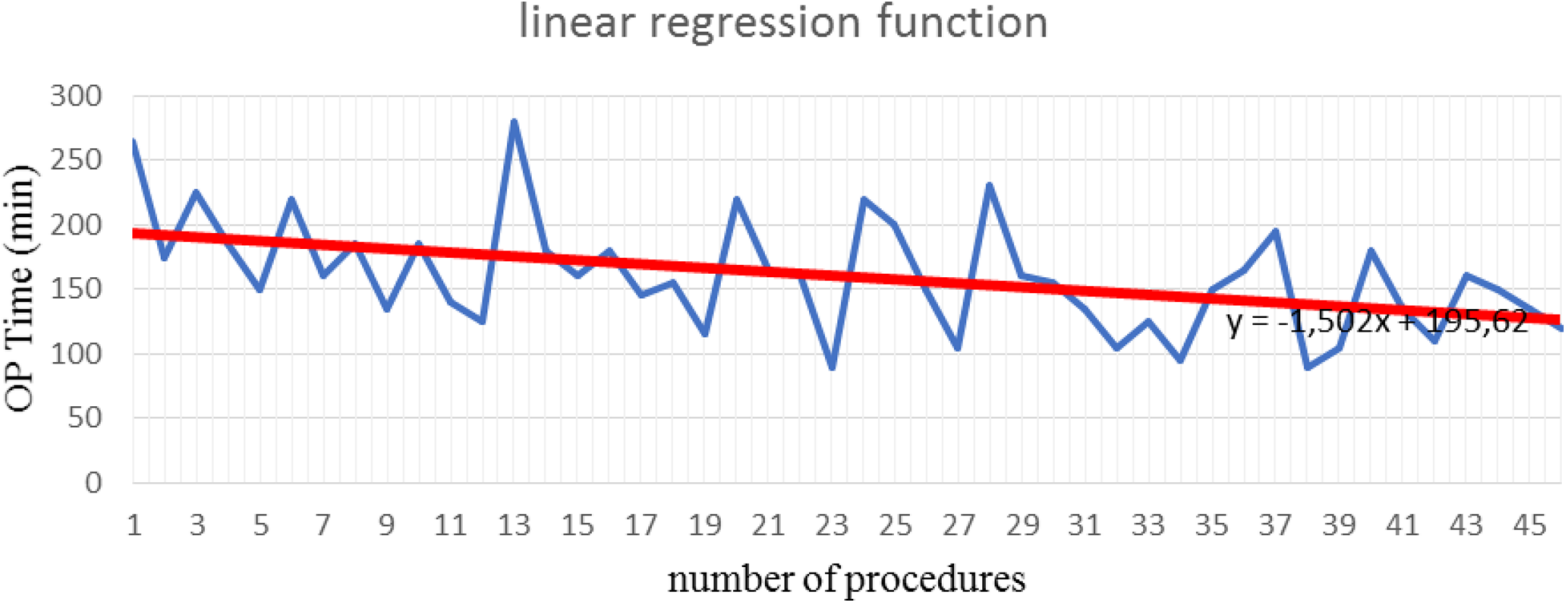
Learning curve after laparoscopic lateral suspension (Max OPTime: 280 min, Min OPTime: 90 min).

The mean operative time was 168.26 min (SD±37.37) for LSC and 160.33 min (SD ±43.91) for LLS. Both procedures were performed with concomitant laparoscopic supracervical hysterectomy. Differences in the mean operative time were not statistically significant. The learning curve after 43 procedures of lateral cervicopexy reduced to 134.7 min (Tables 1, 2). After 43 procedures, the learning curve for laparoscopic lateral suspension was shorter than for laparoscopic sacrocolpopexy (Figures 4, 5).

**Table 1:** OPTime sacrocolpopexy.

Number of procedures	Mean OPTime (min)
0–15	168.00
16–30	164.33
31–43	173.08

**Table 2:** OPTime lateral suspension.

Number of procedures	Mean OPTime (min)
0–15	184.67
16–30	163.33
31–43	134.69

## Discussion

We describe our 1 year experience performing LLS and LSC as a part of our educational program, implemented in the Department of Gynecology, Endocrinology and Gynecologic Oncology at Pomeranian Medical University, Szczecin, Poland.

Two surgical techniques were presented for our urogynecology fellow who had previous experience in laparoscopy. Our aim was to observe the learning curve with regard to approaches.

Learning curves are valuable in surgical training and can guide the development of effective training programs for surgical residents and fellows. We selected following criteria for evaluation of the learning curve: operation time, perioperative complications, and clinical outcomes.

The duration of the operation is often considered a descriptive parameter in evaluation of the learning curve. In our study, during the first 15 operations, the mean duration of procedure (mean OPTime) for laparoscopic promontofixation and for lateral suspension was 168 and 184 min, respectively. Over 30 procedures, the mean OPTime was 164 and 163 min, respectively. Consequently, we noticed that after 30 first procedures of LLS, the operative time was significantly reduced, so this could be the turning point for this surgical procedure. The authors reported the mean total operative time of LSC ranged around 172–250 min, but the same notable decrease after the first 30 cases (16, 17). Mustafa et al. declined plateau after 15 cases, but experience of surgeons was not mentioned. This study also emphasized that operative time does not reflect the surgeon proficiency, with getting experience surgeon will choose more complicated cases. The authors say that lower plateau of learning curve may be explained by new approaches they proposed for laparoscopic sacrocolpopexy (18).

Moreover, above-mentioned studies used technical modifications of laparoscopic sacrocolpopexy including different place of suturing the mesh, various form of meshes or performing concomitant surgical procedures at the same time, which certainly affect at the time of the operation and reliability of results (19).

In vaginal reconstructive surgery, according to Wu et al., 37–47 procedures needed to be performed to gain necessary proficiency with good surgical success and operative time (20).

To train medical professionals in laparoscopy, simulation-based training is commonly used. These simulations involve using laparoscopic models or virtual reality simulators that replicate the conditions of performing surgery. There are various types of laparoscopic training models, including box trainers, virtual reality simulators, animal tissue–based models, and cadaveric training.

Virtual reality simulators are a valuable resource for improving the skills and shortening the learning curve for laparoscopic procedures (21). Benefits of such laparoscopy training include giving learners the opportunity to practice techniques, improve hand–eye coordination, familiarize themselves with laparoscopic instruments, and develop critical skills before operating on real patients (22).

Simulation-based training allows medical professionals to make mistakes in a safe environment and receive feedback from experienced instructors. This type of training can enhance the proficiency and confidence of surgeons, ultimately leading to better patient outcomes during real surgical procedures (23).

Some studies indicated the role of structured curriculums for laparoscopic training, especially in the field of urology (24).

Unfortunately, there is still the lack of training models for laparoscopic urogynecologic skills. In our study, we present the unique setting dedicated for urogynecologic training.

Perioperative complications and quality of life are also important to evaluate the learning curve. According to our study, complication rates were low. The most frequent complication of LSC is *de novo* overactive bladder (OAQ). Data show 13% of its occurrence (19). We have found a negligible rate of three new *de novo* OAB symptoms in LLS (9.3%). Erosion can be present in up to 15% (25, 26); we had no vaginal mesh exposure in our follow-up.

Lateral suspension of the cervix may lead to anterior displacement of the physiological vaginal axis, which may predispose to the occurrence of rectocele in the future. This can probably be avoided by attaching the tape without tension. We did not observe any increased prevalence of the posterior compartment prolapse *de novo* in both groups.

Conversion to laparotomy was necessary in three women: two patients in the sacropexy group and one in the lateral suspension group. These conversions were due to previous postoperative adhesions. Intraoperative complications included bladder injury in two patients after laparoscopic lateral suspension surgery. No other complications were recorded. The difference in the rates of perioperative complications was not statistically significant, which means that throughout the process of reaching the learning curve plateau, treatments performed were characterized by a similar safety rate. These results were confirmed by other authors (16–18, 25–26).

Patient's satisfaction was assessed with the use of a validated PFDI-20 questionnaire (Table 3). Analysis of the quality of life with a validated questionnaire (PFDI-20) after both procedures showed its significant improvement mainly in terms of symptoms. Data demonstrated that the combination of standardized questionnaires with physical examination can help obtain a comprehensive picture of a patient's symptoms, the degree of prolapse, and the effects on daily life (27–29).

**Table 3:** Evaluation of symptoms by using PFDI-20.

Symptoms	LSC *n* (%) 43		*p*	LLS *n* (%) 46		*p*	*p* LLS/LSC
	Preoperative	Postoperative		Preoperative	Postoperative		
Bulging	43 (100)	11 (25.58)	<0.005	42 (91.30)	4 (8.7)	<0.005	>0.005
Urinary urgency wet	24 (55.81)	7 (16.28)	<0.005	26 (56.52)	9 (19.56)	<0.005	>0.005
Urinary frequency	24 (55.81)	7 (16.28)	<0.005	29 (63.04)	10 (21.73)	<0.005	>0.005
OAB	16 (37.21)	4 (9.30)	0.05	23 (50)	6 (13.04)	0.005	>0.005
UI	11 (25.58)	7 (16.27)	0.78	6 (13.04)	2 (4.35)	0.08	>0.005
Constipation	27 (62.79)	12 (27.90)	0.02	27 (58.69)	22 (47.82)	0.187	>0.005

UI, urinary incontinence.

Veit-Rubin et al. declared that 85% of patients had satisfaction of operation, which was similar to our results (30). Our results showed alleviation of such symptoms like bulging, urinary urgency wet, and urinary frequency that lead to active lifestyle and improved physical wellbeing. Women experienced increased comfort and relief from discomfort or pressure in the pelvic region (Table 4).

**Table 4:** Anatomical and functional outcome.

	Laparoscopic sacrocervicopexy	Laparoscopic lateral suspension
Patients	43/89 (48.3%)	46/89 (51.7%)
Apical compartment success rate (%)	90.7%	89.1%
Anterior compartment success rate (%)	88.37	91.3
Complications	One severe back pain	Two bladder injuries
Mean blood loss (ml)	100	90
Operative time (min)	168.26	160.33

Constipation remains the most frequent symptom after POP surgery. The reason for that is posterior mesh placement which reduces posterior pelvic place and may lead to bowel symptoms (31). Teleman et al. emphasize that questionnaires do not help in differentiating weather digestive symptoms relate to POP or other pathologies such as functional gastrointestinal disorders detrusor instability, urethral obstruction, or exaggerated fluid intake (32).

This study has potential limitations. First, the results were based on a single institution experience with only one surgeon and no control group, which may limit the ability to generalize results. Second, we did not include the correlation of complications and failures with surgeon experience. Although we report the 12-month follow-up data, in POP surgery, this can be considered relatively short. A long-term follow-up is needed to draw firm conclusions.

To conclude, we are convinced that specific urogynecologic equipment could be used to assess novice laparoscopic trainees across different specialties and help them acquire laparoscopic competencies prior to supervised surgery. Structured training programs can help surgeons learn and perform laparoscopic procedures properly. However, these should be well designed and strictly supervised by experienced surgeons.

Laparoscopic lateral suspension has shown comparable efficacy and success rates to sacrocolpopexy in treating POP (33). According to our data, LLS can be included in fellowship training because it is safer, easier to perform, and has faster learning curve than laparoscopic sacrocolpopexy. To our knowledge, there is no published report concerning a learning curve for laparoscopic lateral suspension. We believe that our findings would promote the technique and provide significance when designing surgical training programs.

## Data Availability

The raw data supporting the conclusions of this article will be made available by the authors, without undue reservation.
